# Protective Role of α-Calcitonin Gene-Related Peptide in Cardiovascular Diseases

**DOI:** 10.3389/fphys.2019.00821

**Published:** 2019-07-02

**Authors:** Ambrish Kumar, Jay D. Potts, Donald J. DiPette

**Affiliations:** ^1^Department of Cell Biology and Anatomy, School of Medicine, University of South Carolina, Columbia, SC, United States; ^2^Department of Internal Medicine, School of Medicine, University of South Carolina, Columbia, SC, United States

**Keywords:** heart failure, calcitonin gene-related peptide (CGRP), neuropeptide, cardiovascular diseases, hypertension, myocardial infarction

## Abstract

α-Calcitonin gene-related peptide (α-CGRP) is a regulatory neuropeptide of 37 amino acids. It is widely distributed in the central and peripheral nervous system, predominantly in cell bodies of the dorsal root ganglion (DRG). It is the most potent vasodilator known to date and has inotropic and chronotropic effects. Using pharmacological and genetic approaches, our laboratory and other research groups established the protective role of α-CGRP in various cardiovascular diseases such as heart failure, experimental hypertension, myocardial infarction, and myocardial ischemia/reperfusion injury (I/R injury). α-CGRP acts as a depressor to attenuate the rise in blood pressure in three different models of experimental hypertension: (1) DOC-salt, (2) subtotal nephrectomy-salt, and (3) L-NAME-induced hypertension during pregnancy. Subcutaneous administration of α-CGRP lowers the blood pressure in hypertensive and normotensive humans and rodents. Recent studies also demonstrated that an α-CGRP analog, acylated α-CGRP, with extended half-life (~7 h) reduces blood pressure in Ang-II-induced hypertensive mouse, and protects against abdominal aortic constriction (AAC)-induced heart failure. Together, these studies suggest that α-CGRP, native or a modified form, may be a potential therapeutic agent to treat patients suffering from cardiac diseases.

## Introduction

**Figure 1:**
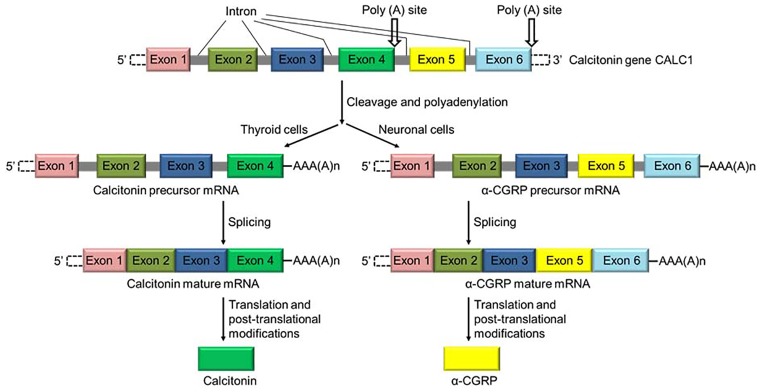
Schematic diagram showing the synthesis of calcitonin and α-CGRP from a common gene CALC-I. Calcitonin gene CALC-I undergoes alternative splicing and forms protein calcitonin in thyroid C cells, and α-CGRP in the sensory neurons.

**Figure 2:**
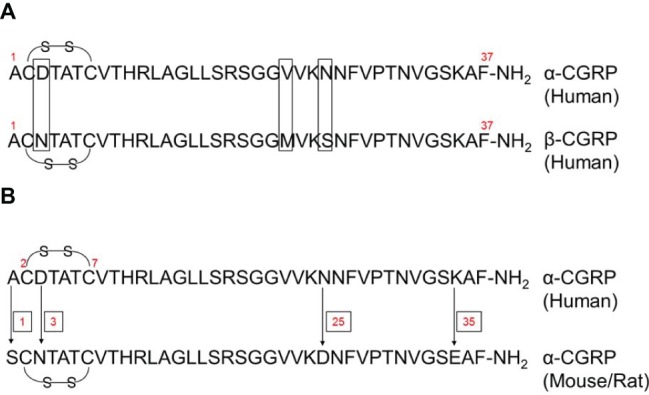
Amino acid sequence of human α- and β-CGRPs, and rodent α-CGRP. **(A)** Human α- and β-CGRPs have a disulfide bridge (-S-S-) at cys2 and cys7, and amidated phenylalanine at C-terminus. Both forms of CGRP share >90% homology, and differ by three amino acids at positions 3, 22, and 25 (as shown by boxes). **(B)** Amino acid sequence of human and rodent (mouse/rat) α-CGRPs. Human and rodent (mouse/rat) α-CGRPs differ at amino acid residues 1, 3, 25, and 35 (as shown by arrows). Both have a disulfide bridge (-S-S-) at cys2 and cys7, and amidated phenylalanine at C-terminus.

α-Calcitonin gene-related peptide (α-CGRP) is a regulatory neuropeptide that belongs to the calcitonin/CGRP peptide family that includes adrenomedullin (ADM), amylin, and calcitonin (Breimer et al., 1988). α-CGRP is generated from the tissue-specific alternative splicing of the primary transcript of the calcitonin gene CALC-I (Amara et al., 1982; Rosenfeld et al., 1983). In thyroid C cells, the CALC-I gene product containing exon 4 forms calcitonin, whereas the CALC-I gene product containing exons 5 and 6 forms α-CGRP in sensory neurons (Figure 1). Peptide α-CGRP is a single polypeptide of 37 amino acids containing a disulfide bridge between positions 2 and 7 and a phenylalanine amide residue at the C-terminal. Another distinct homologue of the CGRP is also known and termed as β-CGRP. However, β-CGRP is encoded from a separate CALC-II gene located on chromosome 11 in humans, and is not produced by alternative splicing of the gene (Steenbergh et al., 1985; Alevizaki et al., 1986). α-CGRP is synthesized in specific regions of the central and peripheral nervous systems, particularly in the sensory neurons of the dorsal root ganglion (DRG). β-CGRP is mainly expressed in the gut, intestine, pituitary gland, and immune cells such as T cells (Petermann et al., 1987; Mulderry et al., 1988; Xing et al., 2000). The α- and β-forms of the CGRP are highly conserved across species and share >90% homology; however, both forms differ by three amino acids in human (Figure 2A; Steenbergh et al., 1986). The biological action of CGRP isoforms is overlapping; moreover, α- and β-CGRPs exerts distinct hemodynamic and gastric effects in humans, respectively (Shekhar et al., 1991; Dubois-Rande et al., 1992). Compared to β-CGRP, α-CGRP has a markedly greater activity in the regulation of cardiovascular functions (Juaneda et al., 2000; Russell et al., 2014).

**Figure 3:**
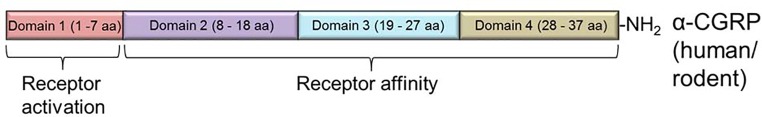
Domain structure of α-CGRP neuropeptide in rodent and human. The peptide is composed of four domains (Domain 1 to Domain 4). Domain 1 is essential for the CGRP receptor activation, while Domains 2–4 are necessary for receptor affinity.

Human and rodent (mouse/rat) α-CGRPs differ at four amino acid positions – 1, 3, 25, and 35 (Figure 2B); nevertheless, both share identical biological activities. Structurally, α-CGRP is composed of four domains (Figure 3):

1. Domain 1: N-terminus Domain 1 spans from amino acid residues 1 to 7 and forms a loop/ring-like structure. NMR studies suggest that the cysteine at positions 2 and 7 forms a disulfide bridge (Cys_2_-Cys_7_) (Breeze et al., 1991), and it has been shown that loss of the disulfide bond abolishes all the biological activity of the α-CGRP peptide. Domain 1 of α-CGRP binds to the transmembrane domain of the receptor and is essential for receptor activation. The N-terminal truncated peptide, CGRP_8–37_, acts as a CGRP receptor antagonist that has a high affinity for the CGRP receptor but lacking any biological activity (Chiba et al., 1989).
2. Domain 2: Domain 2 consists of an α-helix and spans the peptide region from amino acids 8-18. Deletion of this region decreases the affinity of the ligand α-CGRP to its receptor by 50- to 100-fold (Rovero et al., 1992).
3. Domain 3: Domain 3 of α-CGRP spans from residues 19-27 and can form either a β- or γ-twist (Conner et al., 2002) and may make additional receptor contacts or may stabilize an appropriate conformation of amino acids 28-37.
4. Domain 4: This C-terminus region of the peptide lies at residues 28-37 and contains two turn regions which form a putative binding epitope. This C-terminal region of the α-CGRP interacts with N-terminal region of the CGRP-receptor (Carpenter et al., 2001).

Together, biological assays and deletion studies reveal that the amino acids 8-37 (Domain 2 to Domain 4) are required for the affinity of the peptide to its receptor, and the N-terminal Domain 1 of α-CGRP is essential for the receptor activation.

## Calcitonin Gene-Related Peptide Receptor

**Figure 4:**
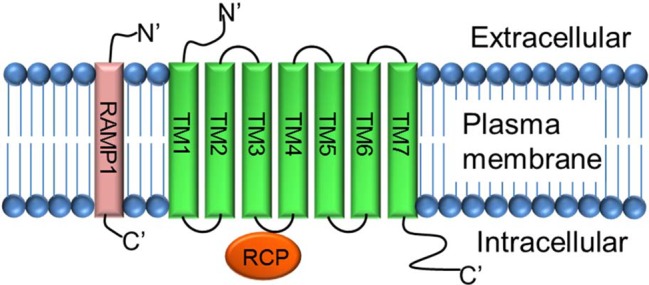
Structure of CGRP receptor. CGRP receptor is a complex heterotrimeric G-protein coupled receptor and requires three protein components: (1) calcitonin receptor-like receptor (CLR; a seven transmembrane protein TM1–TM7), (2) receptor activity modifying protein-1 (RAMP-1; a single transmembrane protein), and (3) receptor component protein (RCP; a small intracellular protein).

CGRP mediates its action through a complex heterotrimeric CGRP receptor that is composed of three proteins: (1) calcitonin receptor-like receptor (CLR, also known as CRLR), (2) receptor activity modifying protein 1 (RAMP 1), and (3) receptor component protein (RCP) molecule (Figure 4).

The calcitonin receptor-like receptor (CLR) is the ligand-binding portion of the receptor and belongs to the class B “secretin-like” family of the G-protein-coupled receptor (GPCR) (Lin et al., 1991; Barwell et al., 2012). The CLR receptor component possesses seven transmembrane spanning domains (TM1–TM7), a N-terminus extracellular domain, and an intracellular C-terminus (Aiyar et al., 1996). RAMP1 is a single transmembrane-spanning domain protein that has a long extracellular N-terminus of 100 amino acids and a short intracellular C-terminal tail of ~10 amino acids (McLatchie et al., 1998). Heterodimerization of CLR and RAMP1 (CLR/RAMP1) is required for transport of the CLR as a mature glycoprotein from the endoplasmic reticulum/Golgi apparatus to the plasma membrane (Hilairet et al., 2001). Deletion of amino acid residues 91-94, 96-100, and 101-103 totally inhibits the production of cAMP, and substitution of five amino acids at C-terminal of RAMP1, next to transmembrane domain, interrupts dimerization of CLR and RAMP1, and decreases receptor functions (Kuwasako et al., 2003). RCP is a small (~17 kDa) intracellular peripheral membrane protein that is associated with the cell membrane through ionic interactions (Evans et al., 2000). The direct interaction of RCP to the CGRP receptor CLR was verified by immunoprecipitation experiments where RCP was co-immunoprecipitated with the CLR/RAMP complex. Further analyses using a yeast two-hybrid assay and co-immunoprecipitation experiments confirmed that RCP directly interacts with the second intracellular cytoplasmic loop (ICL2) of CLR (Egea and Dickerson, 2012). Studies carried out in NIH3T3 cells expressing a functional CGRP receptor complex CLR-RAMP1 showed that depletion of RCP, using an antisense RNA approach, had no effect on the binding of the α-CGRP ligand to its receptor CLR. However, loss of RCP significantly abolished the α-CGRP-mediated signaling as demonstrated by a significant decrease in the production of cAMP (Evans et al., 2000). Thus, RCP links the receptor CLR to an intracellular G protein-mediated signaling pathway that increases intracellular cAMP levels by activating adenylate cyclase. These studies confirm that CLR, RAMP1, and RCP are required to form a truly functional α-CGRP receptor.

The binding of α-CGRP to its receptor is a two-step process which has been termed the “two-domain model” (Hoare, 2005). This model suggests that the C-terminus of the α-CGRP initially interacts with the N-terminus of the extracellular domain of CLR. Next, the N-terminus of α-CGRP binds to the transmembrane domain and extracellular loop region of the CLR that triggers receptor activation (Kuwasako et al., 2003).

## α-Calcitonin Gene-Related Peptide and Its Receptor Distribution and Cell Signaling

α-CGRP and its receptor are widely distributed in the central and peripheral nervous system and the cardiovascular system (Mulderry et al., 1985). In the peripheral nervous system, the dorsal root ganglia (DRGs) are the prominent sites of α-CGRP synthesis. The DRG contains the cell bodies of sensory nerves that terminate peripherally on blood vessels and centrally in laminae I/II of the spinal cord. A dense perivascular α-CGRP-containing neural network is seen around the blood vessels in all vascular beds (Uddman et al., 1986). It is thought that circulating α-CGRP is largely released from these perivascular nerve terminals. The presence of α-CGRP receptor has been reported in the endothelium, media and intima of vessels, veins, and in multiple tissues and organs.

**Figure 5:**
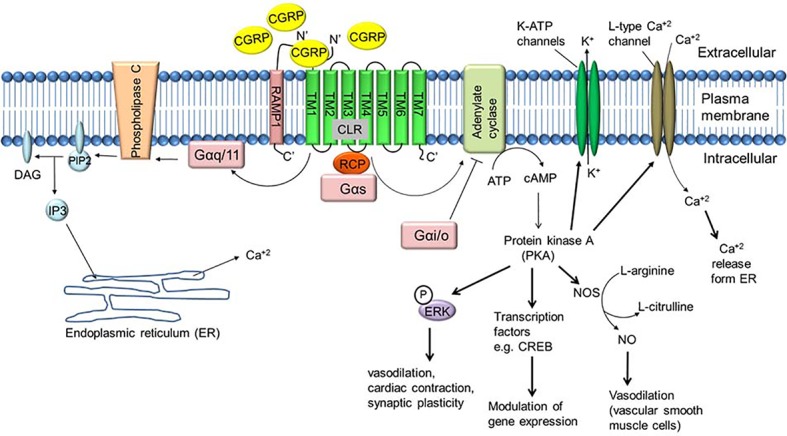
α-CGRP-mediated intracellular signaling. α-CGRP elevates intracellular cAMP level through CLR/RAMP1, G-protein Gα_s,_ and adenylate cyclase (AC) activation. Elevated level of cAMP activates protein kinase A (PKA) and increases various downstream signaling molecules, including the potassium-sensitive ATP channels (K-ATP channels), L-type Ca^+2^ channel, ERK, and cAMP response element-binding protein (CREB). In addition, α-CGRP signaling is also able to act through other G-proteins such as Gα_i/o_ and Gα_q/11_. CGRP through Gα_q/11_ stimulates the IP3-PLC pathway leading to an increase in intracellular Ca^+2^ concentrations. DAG, diacylglycerol; IP3, inositol triphosphate; PIP2, phosphatidylinositol 4,5-bisphosphate.

Several lines of evidence suggest that binding of α-CGRP to its receptor CLR activates a variety of signaling pathways (Figure 5). In most instances, binding of α-CGRP to the CLR/RAMP1 receptor activates adenylate cyclase through the G-protein Gα_s_ that in turn elevates intracellular cAMP level. Increased levels of CGRP-induced cAMP activate protein kinase A (PKA) and increase various downstream signaling molecules, including the potassium-sensitive ATP channels (K-ATP channels), L-type Ca^+2^ channels, ERK, and cAMP response element-binding protein (CREB). In human embryonic kindney-296 cells, α-CGRP activates ERK by the PI3K-PKA pathways and p38 through PKA signaling. These downstream signaling pathways regulate CGRP actions, including vasodilation, cardiac contraction, and synaptic plasticity (Nelson et al., 1990; Hong et al., 1996; Anderson and Seybold, 2004). In addition, CGRP signaling is also able to act through other G-proteins such as Gα_i/o_ and Gα_q/11_. It has been reported that activity of CGRP through Gα_q/11_ stimulates the IP3-PLC pathway leading to an increase in intracellular Ca^+2^ concentrations, thereby activating protein kinase C (PKC) (Aiyar et al., 1999). Disa et al. (2000) reported that addition of pertussis toxin, which blocks the actions of Gi/Go-type G proteins, blocks CGRP-mediated activation of JNK (c-Jun N-terminal kinase) in the neuroblastoma cell line SK-N-MC cells (Disa et al., 2000). Another study demonstrated that administration of pertussis toxin abolished CGRP-induced stimulation of Ca^+2^ currents in rat nodose ganglion neurons (Wiley et al., 1992). These data support the involvement of Gα_i/o_-signaling mediated by CGRP and CGRP receptors. Finally, the CGRP receptor also has an inhibitor role when it couples to Gα_i/o,_ reducing adenylate cyclase (AC) activity and hence reducing intracellular level of cAMP and decreasing protein kinase A (PKA) activity.

## Regulation of α-Calcitonin Gene-Related Peptide Expression and Release From Sensory Nerves

α-CGRPs containing nerve fibers are components of the primary afferent nervous system, comprising principally of myelinated (Aδ)- and unmyelinated (C)-fiber nerves that respond to chemical, thermal, and mechanical stimuli. Although these nerves have traditionally been thought to “sense” stimuli in the periphery and transmit the information centrally, it is now well established that they have an efferent function as well. It is clear that DRG neuron-derived peptides are released at peripheral sensory nerve terminals in the absence of any stimulation. The continuous expression and release of peptides from DRG neurons may reflect a paracrine function implying that these neurons participate in the continuous regulation of blood flows and other tissue activities.

Various stimuli have been identified that regulate the expression of α-CGRP in different cell types. Multiple chemical and physiological factors such as nerve growth factor (NGF), angiotensin II (Ang-II), brain-derived neurotrophic factor (BDNF), bradykinins and prostaglandins, endothelin, the renin/angiotensin/aldosterone system, and tissue inflammation are known to increase α-CGRP synthesis (Lindsay, 1988; Supowit et al., 1995b, 2001; Freeland et al., 2000; Salio et al., 2007). Freeland et al. (2000) used a primary culture of adult DRG neurons and showed that NGF treatment phosphorylates the CRBP transcription factor that in turn activates the CGRP promoter, and thus increases CGRP expression, *via* the Ras/Raf/MEK-1/p42/p44 MAPK pathway (Freeland et al., 2000). Supowit et al. (2001) later demonstrated that an intraperitoneal injection of NGF stimulates synthesis and release of CGRP in DRG sensory nerves in spontaneously hypertensive rats (SHRs) (Supowit et al., 2001). Following synthesis, α-CGRP remains stored in the large vesicles within the sensory nerve terminal that, after neuronal depolarization, is released from the neuronal terminal *via* calcium-dependent exocytosis. The release of α-CGRP from the sensory nerves is increased by capsaicin, low pH, bradykinins and prostaglandins, Ang-II, and norepinephrine. In contrast, steroids and mediators such as opioids inhibit α-CGRP release (Meng et al., 2007). In human plasma, α-CGRP has very short half-life (*t*_1/2_ = ~5 min) (MaassenVanDenBrink et al., 2016), and it has been suggested that the endopeptidases endothelin-converting enzyme-1 (ECE-1) and insulin-degrading enzyme (IDE) are responsible for the degradation of α-CGRP (Kim et al., 2012; Hartopo et al., 2013). Using liquid chromatography-tandem mass spectrometry (LC-MS/MS), peptidomics and genetic approaches have demonstrated that IDE primarily cleaves α-CGRP at the Ser_17_-Arg_18_ and Asn_26_-Phe_27_ sites. LC-MS/MS analysis of mouse spinal cords detected a total of 10 CGRP fragments with the major fragments consisting of two N-terminal fragments, CGRP_1–17_ and CGRP_1–26_, and two C-terminal fragments, CGRP_18–37_ and CGRP_26–37_. Similar cleaved peptide fragments have been detected by LC-MS/MS when CGRP_1–37_ was incubated either with the mouse spinal cord lysate or plasma under *in vitro* conditions. The identification of these four peptide fragments indicates that full-length CGRP_1–37_ has two major cleavage sites, Ser_17_-Arg_18_ and Asn_26_-Phe_27._ In order to identify the CGRP-degrading enzyme, a series of biochemical assays were performed and it was ultimately determined that IDE is the only enzyme that cleaves CGRP_1–37_ at Ser_17_-Arg_18_ and Asn_26_-Phe_27._ The observed lower level of CGRP_1–37_ in IDE^+/+^ mice, in comparison to IDE^−/−^ mice, further established IDE as an endogenous regulator of α-CGRP (Kim et al., 2012).

## Cardiovascular Actions of α-Calcitonin Gene-Related Peptide

**Figure 6:**
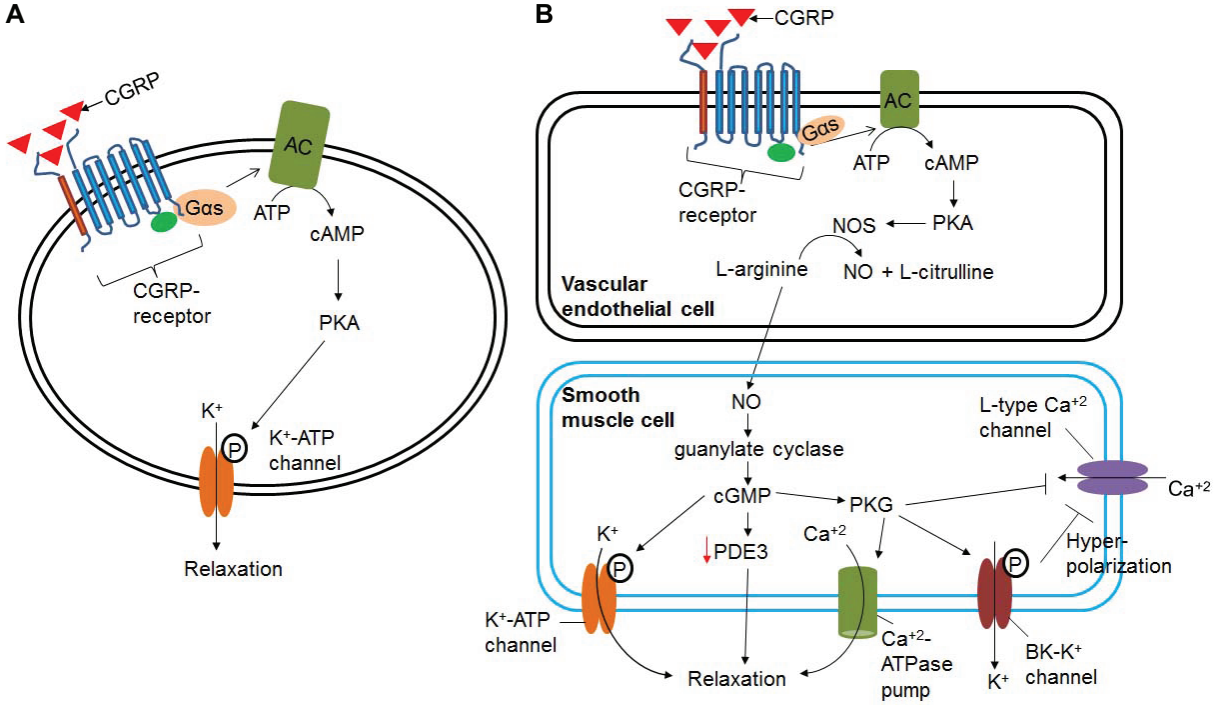
Signaling pathways involved in α-CGRP-induced vasodilation. **(A)** In NO- and endothelium-independent pathway, binding of α-CGRP to its receptor, CLR, increases intracellular cAMP level through G-protein coupled adenylate cyclase (AC). The increased cAMP level activates protein kinase A (PKA), and that in turn phosphorylates and opens K^+^-ATP channels and thus relaxation of vascular smooth muscle cells. **(B)** NO- and endothelium-dependent vasodilatation to α-CGRP. α-CGRP through its receptors activates eNOS enzyme *via* Gαs-adenylate cyclase-cAMP-PKA pathway, and thus increases the synthesis of NO in the endothelial cells. From the endothelial cells NO freely diffuses to the smooth muscle cells activating guanylate cyclase and increasing intracellular cGMP level. These events activate and phosphorylate K-ATP channel that leads to relaxation of smooth muscle cells. cGMP has also been reported to mediate its vasodilation action through cGMP-dependent protein kinase G (PKG), phosphodiesterases (PDEs), and cGMP-gated ion channels. NO/cGMP/PKG signaling pathway efflux intracellular potassium through BK-K channel, promotes hyperpolarization of the plasma membrane, and thereby decreases influx of calcium through L-type Ca^+2^ channels. PKG also can efflux intracellular calcium through the plasma membrane Ca^+2^-ATPase pump. This, in turn, decreases the contractile state of smooth muscle cells.

α-CGRP is the most potent vasodilator known till date and is ~1,000 times more potent than acetylcholine, substance P, and 5-hydroxytriptamine (Brain et al., 1985). An intradermal injection of 10 pmol α-CGRP raised local blood flow, as assessed by laser Doppler flow meter, and induced an erythema in human skin (Brain et al., 1986). It has positive chronotropic, inotropic, and pro-hypertrophic effects in humans (Franco-Cereceda et al., 1987; Gennari et al., 1990; Bell et al., 1995; Al-Rubaiee et al., 2013). The inotropic activity of α-CGRP is mediated by the cAMP/PKA or PKC signaling pathways. It has been shown that α-CGRP dilates multiple vascular beds, including coronary vasculature (Gulbenkian et al., 1993). In addition, α-CGRP administration decreases blood pressure in normotensive and hypertensive animals and humans (DiPette et al., 1989; Dubois-Rande et al., 1992; Supowit et al., 1993). The α-CGRP-mediated reduction in BP is caused by the dilation of peripheral arteries. The biological activity of α-CGRP, i.e., vasodilation/vasorelaxation, can be mediated *via* NO- and endothelium-independent or NO- and endothelium-dependent pathways (Figure 6). The vasodilation activity of α-CGRP through NO- and endothelium-independent pathway is observed in the majority of tissues (e.g., rat perfused mesentery, porcine coronary artery, and cat cerebral artery) where binding of α-CGRP to its receptor, CLR, increases intracellular cAMP level through G-protein coupled adenylate cyclase (Edvinsson et al., 1985; Han et al., 1990; Yoshimoto et al., 1998). The increased cAMP level activates protein kinase A (PKA), that in turn phosphorylates and opens K^+^-ATP channels and thus relaxation of vascular smooth muscle cells (Figure 6A) (Nelson et al., 1990). However, there is scant evidence that vasodilation evoked by α-CGRP is mediated, in part, by nitric oxide (NO) release (Figure 6B; Gray and Marshall, 1992a,b). It has been hypothesized that various vascular beds differ in their dilatory response to α-CGRP due to the endothelium releasing α-CGRP (Bergdahl et al., 1999). The vasorelaxation activity of α-CGRP in the rat aorta, human internal mammary artery, and rat pulmonary artery is reported to be mediated by NO- and endothelium-dependent mechanism (Brain et al., 1985; Gray and Marshall, 1992a; Raddino et al., 1997; Wisskirchen et al., 1998). Addition of the inhibitors of NO synthase (NOS) enzyme attenuates the relaxation action of α-CGRP in the rat aorta (Gray and Marshall, 1992b). Gray and Marshall (1992a) further demonstrated that, in the rat aorta, human α-CGRP caused an increase in the level of cAMP and cGMP only in the presence of an intact endothelium. In the absence of an intact endothelium, α-CGRP produced no significant alterations in either cGMP or cAMP, and no subsequent relaxation of the rat aorta (Gray and Marshall, 1992a). At the cellular level, NO is produced from L-arginine *via* NOS enzymes – eNOS, iNOS, and nNOS. It has also been shown that cAMP can stimulate eNOS activity, and thus increases synthesis and release of NO (Ferro et al., 1999). Another study further demonstrated that the catalytic subunit of PKA can activate and phosphorylate eNOS (Butt et al., 2000). These lines of evidence suggest that NO- and an intact endothelial-dependent pathway are required to elicit α-CGRP-induced relaxation of smooth muscle cells (Figure 6B). In endothelial cells, α-CGRP works through its receptor (CLR) and Gαs protein, *via* activation of adenylate cyclase (AC), which enhances intracellular levels of cAMP. Increased cAMP levels activate and phosphorylate eNOS *via* PKA activation, thus increasing synthesis and release of NO. NO freely diffuses into the surrounding smooth muscle cells and activates guanylyl cyclase. Activated guanylyl cyclase increases the intracellular level of cGMP that in turn activates and phosphorylates the K-ATP channel, and thus relaxes smooth muscle cells (Figure 6B). In addition to ATP-sensitive K^+^ channel, cGMP has also been reported to mediate its vasodilation/vasorelaxation action through cGMP-dependent protein kinase G (PKG), phosphodiesterases (PDEs), and cGMP-gated ion channels (Francis et al., 2010; Takimoto, 2012; Inserte and Garcia-Dorado, 2015). cGMP-dependent protein kinase G (PKG) phosphorylates a variety of proteins including cardiac troponin I, L-type Ca^+2^ channel, and titin, and accelerates cGMP-mediated relaxation of cardiac myocytes (Takimoto, 2012). PKG promotes, through phosphorylation/activation, opening of large-conductance calcium-activated potassium channels (BK-K^+^ channels), and thereby loss of intracellular potassium. These events hyperpolarize the plasma membrane and decrease calcium influx through L-type calcium channels (Zhou et al., 1996). PKG also reduces intracellular Ca^+2^ concentrations by stimulating Ca^+2^-ATP-dependent calcium efflux *via* the plasma membrane Ca^+2^-ATPase pump, thereby causing vascular smooth muscle cells to relax (Francis et al., 2010). Hence, NO/cGMP/PKG signaling pathway lowers intracellular free calcium and decreases the contractile state of smooth muscle cells. The presence of large-conductance calcium-activated potassium channels (BK-K^+^ channels) in pial arteries suggests that the NO/cGMP-mediated signaling pathway may be involved in CGRP-induced vasodilation (Hong et al., 1996). It has also been shown that NO-induced elevation of cGMP enhances the vasorelaxant responses by impairing cAMP hydrolysis by phosphodiesterase type 3 (PDE3) (Figure 6B; Stangherlin et al., 2011). Eberhardt et al. (2014) reported that NO activating *via* a neuroendocrine signaling pathway HNO-TRPA1-CGRP could also induce vasodilation. Studies have demonstrated that, in smooth muscle cells, NO reacts with another gasotransmitter, hydrogen sulfide (H_2_S), and produces a redox molecule, nitroxyl (HNO). HNO activates the transient receptor potential channel (TRPA1) and, thus, increases calcium influx. As a result, CGRP is released, which in turn induces local and systemic vasodilation (Eberhardt et al., 2014).

Several lines of evidence suggest that α-CGRP protects the heart from damage due to hypertension, myocardial infarction, and heart failure (Li et al., 2013), and cardiac and cerebral ischemia (Schebesch et al., 2013). Both *In vitro* and *in vivo* studies revealed that α-CGRP protects cardiomyocytes from apoptotic cell death by activation of the PI3K-Akt and Erk 1/2 kinase pathways which in turn activate cell survival pathways (Schaeffer et al., 2003; Sueur et al., 2005). α-CGRP has also been shown to regulate cardiomyocyte survival by inhibiting oxidative stress by the PI3K-AKT and MAPK signaling pathways (Umoh et al., 2014). α-CGRP was demonstrated to protect H9c2 cardiac cells from hypoxia-induced inflammation and apoptosis by modulating NO production (Duan et al., 2016). In these hypoxia-induced H9c2 cells, α-CGRP treatment significantly decreased the levels of pro-inflammatory molecules (IL-6 and TNF-α) as well as apoptotic proteins (caspase-3, and cytochrome-C), and enhanced the expression of basal and phosphorylated eNOS. The role of α-CGRP in various animal models of cardiovascular diseases has been summarized in Supplementary Table S1.

### Heart Failure

After synthesis, α-CGRP remains in large dense vesicles within the sensory nerves that are located in the perivascular layer of coronary blood vessels and between myofibrillar bundles throughout the myocardium and prominently innervate the heart. This distribution makes them perfectly situated to sense and respond to changes in blood pressure, ischemia, and cytotoxic stress in the heart. Therefore, the efferent function of sensory nerves, exerted *via* CGRP, could provide a protective function against the myocardial remodeling and dysfunction present in response to hypertension and heart failure.

Studies from our lab have shown the cardioprotective activity of α-CGRP against heart failure (Li et al., 2010, 2013). Elevated plasma levels of α-CGRP and CGRP receptor have been observed in individuals with heart failure, and an acute intravenous infusion of α-CGRP significantly improves cardiac functional parameters in patients with congestive heart failure (Gennari et al., 1990). In humans, circulating levels of α-CGRP are elevated during the initial and middle stages of heart failure but then significantly decline as heart failure progresses (Dubois-Rande et al., 1992). Studies carried out in a non-ischemic rat model of chronic heart failure (CHF) demonstrated that pressure-overload heart failure, induced by ascending aortic banding, increases the expressions of RAMP1 mRNA and protein in both atria and ventricles (Cueille et al., 2002). Furthermore, α-CGRP protects against cardiac hypertrophy in an isoprenaline-induced model of heart failure (Li et al., 2010). Gangula et al. (2000) reported markedly elevated basal systolic blood pressure and the mean arterial pressure (MAP) in α-CGRP KO mice when compared to WT mice (Gangula et al., 2000). Subsequently, Li et al. (2013) performed a series of experiments in α-CGRP KO mice and validated the cardioprotective role of α-CGRP in heart failure (Li et al., 2013). They observed that α-CGRP KO mice that underwent pressure-overload heart failure, induced by transverse aortic constriction (TAC), had a lower survival rate compared to their TAC-WT counterpart. The survival rate of the TAC-WT animals (28 days) was approximately 95%. In comparison, the survival rate of the TAC α-CGRP KO mice between days 0 and 21 was approximately 80% and decreased dramatically from days 21 to 28 to approximately 50%. Echocardiography data demonstrated that TAC α-CGRP KO mice exhibit increased adverse cardiac remodeling and dysfunction compared to the TAC-WT mice counterparts. TAC procedure further lowers fractional shortening and ejection fraction in α-CGRP KO mice compared to TAC-WT mice. The ratios of heart weight to body weight and lung weight to body weight were higher in TAC α-CGRP KO mice compared to the TAC-WT mice. Moreover, TAC α-CGRP KO mice had an increased left ventricular hypertrophy and displayed greater cardiac fibrosis, apoptosis and necrosis, inflammation, and attenuation of angiogenesis compared to TAC-WT hearts. Together, these studies demonstrated that absence of the α-CGRP gene heightened the adverse cardiac remodeling, dysfunction, and mortality in pressure-overload-induced heart failure.

### Ischemia/Reperfusion Injury

Following acute myocardial infraction, increased immunoreactive α-CGRP levels have been reported in human plasma and nerves (Mair et al., 1990; Roudenok et al., 2001). Myocardial ischemia causes a marked increase in the release of α-CGRP in isolated hearts from guinea pig and rat (Franco-Cereceda, 1988). During cardiac ischemia/reperfusion (I/R) injury, the C-fiber and Aδ-fiber sensory nerves play a possible efferent cardioprotective role that is mediated through the release of α-CGRP (Wang and Wang, 2005). The release of α-CGRP is induced by the activation of the TRPV1 receptor by the stimulation by protons, bradykinin, and prostaglandins (Strecker et al., 2006). A recent study in TRPV1^−/−^ mice showed that transaortic constriction (TAC) surgery enhances the cardiac hypertrophy and cardiac dysfunction in TRPV1-KO mice compared to their wild-type TAC mice. TAC TRPV1^−/−^ mice had developed left ventricular (LV) hypertrophy with increased LV mass, fibrosis, and increased secretion of TNF-α, and interleukin-6. TAC procedure significantly increased α-CGRP release in wild-type mice, but not in TRPV1^−/−^ mice, suggesting that the deteriorated cardiac function after TAC may be due to the decreased CGRP level in absence of TRPV1 (Zhong et al., 2018). Several independent studies demonstrated that α-CGRP significantly reversed myocardial I/R injury and played a role in cardiac preconditioning and remote preconditioning (Wolfrum et al., 2005). α-CGRP was also shown to attenuate I/R injury-induced cardiac mitochondrial dysfunction through inhibition of GSK 3β *via* PI3k-Akt and ERK 1/2 kinase cascades (Schaeffer et al., 2003; Hausenloy and Yellon, 2004).

Mair et al. (1990) reported that intracoronary infusion of α-CGRP delayed the onset of myocardial ischemia in patients with stable angina pectoris (Mair et al., 1990). In addition, exogenous administration of α-CGRP mimics the cardioprotective effect of preconditioning induced by transient ischemia in a Langendorff-perfused rat heart resulting in an increased release of endogenous α-CGRP. However, administration of BIBN4096BS, a potent α-CGRP receptor antagonist, completely abolished the cardioprotection induced by preconditioning and by exogenous α-CGRP (Wu et al., 2001). Huang et al. (2008) used α-CGRP KO mice to study role of α-CGRP in I/R injury by subjecting isolated-perfused hearts from α-CGRP KO and wild-type mice to an I/R protocol by performing 30 min of ischemia followed by reperfusion (Huang et al., 2008). Following I/R injury, hearts from the α-CGRP KO mice exhibited a significant reduction in cardiac performance compared to their WT counterparts. These α-CGRP KO mice with I/R injury showed an elevated level of creatine kinase (a marker for cell death) and malondialdehyde (a marker for oxidative stress-induced lipid peroxidation) in the heart. Therefore, these studies demonstrate that deletion of α-CGRP makes the heart more vulnerable to I/R injury likely due to increased oxidative stress and inflammation. In summary, these studies suggest an important role for α-CGRP in ischemic preconditioning.

### Hypertension

In the hypertensive patients with primary aldosteronism or on a high-salt diet, an increased level of circulating CGRP has been detected (Resnick et al., 1989). Several studies from our laboratory and others have established a direct role for α-CGRP in experimental hypertension (Marquez-Rodas et al., 2006). Our laboratory has demonstrated that α-CGRP acts as a compensatory depressor to attenuate the rise in blood pressure in three different models of experimental hypertension: (1) deoxycorticosterone (DOC)-salt (Supowit et al., 1997), (2) subtotal nephrectomy-salt (Supowit et al., 1998), and (3) L-NAME-induced hypertension during pregnancy (Gangula et al., 1997). A similar role for CGRP has also been shown in a two-kidney one-clip model of hypertension (Supowit et al., 1998), and in chronic hypoxic pulmonary hypertension (Tjen et al., 1992; Bivalacqua et al., 2002). The expression of α-CGRP and α-CGRP receptor is increased in several models of hypertension including an Ang-II model (Li and Wang, 2005).

The level of CGRP in neuronal cells is differentially regulated in several models of hypertension. In the DOC-salt-induced hypertensive rat, elevated levels of immunoreactive CGRP in the spinal cord and α-CGRP mRNA in the DRG were observed (Supowit et al., 1995a). In comparison, decreased levels of immunoreactive CGRP were observed in laminae I and II of the spinal cord, and CGRP mRNA was observed in the DRG in the spontaneously hypertensive rats (SHRs) (Supowit et al., 1993). These results suggest that the lower level of α-CGRP expression in the SHR might contribute to the high blood pressure observed by the relative reduction of vasodilator activity, whereas higher α-CGRP levels in the DOC-salt hypertension animal attenuate the elevated blood pressure by the compensatory vasodilator activity. The latter notion is supported by the fact that in DOC-salt hypertensive rats, the baseline mean arterial pressure was higher than in normotensive rats (175 ± 5 vs. 119 ± 4 mm Hg, *p* < 0.001). This coupled with the fact that intravenous administration of an α-CGRP receptor antagonist (CGRP_8–37_) to DOC-salt rats significantly further increased the mean arterial pressure, (Supowit et al., 1997). Similarly, infusion of the antagonist α-CGRP_8–37_ increased the elevated blood pressure in two other models of experimental hypertension: subtotal nephrectomy (SN)-induced hypertensive rats (Supowit et al., 1998) and N-nitro-L-arginine methyl ester (L-name)-induced hypertension during pregnancy (Gangula et al., 1997). The anti-hypertensive activity of α-CGRP was further confirmed using α-CGRP KO mice (Gangula et al., 2000; Bowers et al., 2005; Supowit et al., 2005). Gangula et al. (2000) reported that basal blood pressure and mean arterial pressure (MAP) were significantly higher in α-CGRP KO mice compared to wild-type mice of a similar strain (Gangula et al., 2000). Another study using a DOC-salt model using α-CGRP KO and WT mice reported that both heart and kidney sections from the α-CGRP KO mice displayed significantly greater cellular damage and had increased levels of pro-inflammatory cytokines and chemokines such as intercellular adhesion molecule-1, vascular adhesion molecule-1, and monocyte chemoattractant protein-1 (Supowit et al., 2005). Compared to the DOC-salt WT mice, oxidative stress measured by urinary microalbumin and isoprostane accumulation were significantly increased in the hypertensive DOC-salt α-CGRP KO mice. These results demonstrated that deletion of α-CGRP makes the heart and kidneys more susceptible to hypertension-induced end organ damage possibly through enhanced oxidative stress and inflammation. These studies further confirm that CGRP found in sensory nerves protects against hypertension-induced heart and kidney damage.

Another study using a conditional deletion of RAMP1 (RAMP1^−/−^) found that RAMP1-KO mice are hypertensive due to the disruption of α-CGRP signaling (Tsujikawa et al., 2007). Administration of α-CGRP caused a potent relaxation of the arteries in WT mice, but failed to do so in RAMP1-KO mice. Furthermore, Ang-II is a vasoconstrictor and is known to induce hypertension in human (Laragh et al., 1972) and rodents; however, transgenic expression of human RAMP1 blunts the Ang-II-induced adverse effects of hypertension in mice (Sabharwal et al., 2010). It is well known that the renin-angiotensin-aldosterone system (RAAS) is involved in the development of hypertension (Deng and Li, 2005). In α-CGRP KO mice, an increase in the activity of the circulating RAAS is observed, which may be the reason for the higher blood pressure in these mice (Li et al., 2004). It has been shown that addition of a sub-depressor dose of α-CGRP significantly reduced the blood pressure in hypertensive rats, induced by Ang-II or norepinephrine (Fujioka et al., 1991). α-CGRP is also known to inhibit the secretion of aldosterone, induced by Ang-II (Murakami et al., 1991). In spontaneously hypertensive rats (SHRs), aldosterone decreases the vasoconstriction response to electric field stimulation in mesenteric arteries by increasing the vasodilatory response to CGRP (Balfagon et al., 2004). These studies confirm that α-CGRP plays a role in maintaining the blood pressure *via* interaction with the renin-angiotensin-aldosterone system. A recent study carried out in vascular smooth muscle cells demonstrated that a disintegrin and metalloproteinase 17 (ADAM17) is involved in the protective effect of α-CGRP against Ang-II-induced inflammation *via* the EGFR-ERK1/2 pathway (Zeng et al., 2018). Moreover, in Ang-II-induced hypertensive mice, increased levels of oxidative stress, apoptosis, and inflammation were observed in the heart and kidney (Aubdool et al., 2017). In these mice, Ang-II upregulates the expression of oxidative stress response proteins, e.g., NADPH detablehydrogenase quinone-1 (NQO1), hypoxia-inducible factor 1α (HIF-α), NADPH oxidase-2 (NOX-2), and heme oxygenase-1 (HO-1), and downregulates the expression of anti-oxidative enzyme glutathione peroxidase-1 (GPX-1) (Aubdool et al., 2017). Ang-II also significantly decreases eNOS expression that in turn causes endothelial dysfunction and vascular hypertrophy (Cai and Harrison, 2000; Aubdool et al., 2017). Finally, Ang-II significantly increases the protein expression of the CGRP receptor RAMP1 in the aorta, mesentery, and heart. These Ang-II adverse effects worsen the cardiac function creating a pathophysiological condition. However, subcutaneous administration of an α-CGRP agonist analog, acylated α-CGRP, significantly attenuated Ang-II-induced adverse effects in heart, kidney, and vessels. The acylated α-CGRP reduced cardiac hypertrophy and remodeling, fibrosis, inflammation and oxidative stress in Ang-II-induced hypertensive mice (Aubdool et al., 2017). These observations from our lab and others provide direct evidence for the involvement of α-CGRP and its receptor in the regulation of blood pressure and their protective functions in hypertension.

## α-Calcitonin Gene-Related Peptide as a Therapeutic Agent

The beneficial protective effects of α-CGRP in various cardiovascular diseases have driven research to develop α-CGRP as a therapeutic agent to treat cardiac diseases. Previous studies showed that administration of α-CGRP to normotensive patients decreased blood pressure in a dose-dependent manner (Franco-Cereceda et al., 1987). In addition, intravenous infusion of α-CGRP for 24 h improved myocardial contractility in patients suffering from congestive heart failure (Gennari et al., 1990). In a similar study, infusion of α-CGRP in patients with heart failure decreased systemic arterial pressure in a dose-dependent manner (Dubois-Rande et al., 1992). These human studies confirm the idea that α-CGRP is a promising therapeutic agent to treat patients suffering from cardiovascular diseases. However, the short half-life of α-CGRP (*t*_1/2_ = 5 min) in human plasma makes it difficult to use in any long-term treatment regimes. Therefore, novel strategies are needed for α-CGRP delivery and synthesis of innovative α-CGRP agonist analogs. These approaches will be helpful to extend the bioavailability of α-CGRP for longer duration of time in plasma. In recent years, the development of α-CGRP agonist with a longer half-life and bioavailability has been developed (Nilsson et al., 2016; Aubdool et al., 2017; Sheykhzade et al., 2018). Aubdool et al. (2017) tested an acylated form of α-CGRP (developed by Novo Nordisk) in rodent models of hypertension and heart failure. They demonstrated that a daily systemic subcutaneous administration of the α-CGRP analog (50 nmol/kg per mouse) reversed the renal, vascular, and cardiac damage caused by Ang-II-induced hypertension or by abdominal aortic constriction (AAC)-induced heart failure. The acylated αCGRP lowered blood pressure, and reduced cardiac fibrosis, oxidative stress, and cardiac hypertrophy in these mice with hypertension and heart failure (Aubdool et al., 2017). This α analog had an extended half-life in serum (~7 h) and exhibited similar pharmacological properties to native αCGRP in isolated human and rodent vasculature. Similar to native α-CGRP, the acylated αCGRP-induced vasodilation was reversed with similar potencies by the addition of a CGRP receptor antagonist BIBN4096BS (Sheykhzade et al., 2018). Novo Nordisk has also developed another α-CGRP agonist, Serinyl-α-CGRP (2–37)-amide, consisting of an albumin-binding fatty acid moiety in the N-terminus. They demonstrated that serinyl-α-CGRP administration for 2 weeks reduced fasting blood glucose levels and fasting insulin levels in type2 diabetic ob/ob mice. These data suggest that serinyl-α-CGRP may have a therapeutic potential for the treatment of type 2 diabetes (Nilsson et al., 2016). Further research into other modifications is ongoing to develop α-CGRP agonist analogs and warranted given the immense therapeutic potential of αCGRP in a variety of human diseases.

It should also be noted that patients experiencing migraine headaches show an increase in serum level of α-CGRP, and that the increase was observed for both episodic and chronic migraine (Russo, 2015). Clinical trials carried out during the past decade have proved that CGRP receptor antagonists are effective for treating migraine, and antibodies to the receptor and CGRP are currently under investigation. Several antagonists of α-CGRP are currently being developed and tested in order to relieve migraine headache in patients (Maasumi et al., 2018). These antagonists either interact with the α-CGRP receptor or bind to α-CGRP directly to inhibit the interaction between α-CGRP and α-CGRP receptor, thus abolishing the biological activities of α-CGRP. Recently, the Food and Drug Administration (FDA) approved three new monoclonal antibodies that target CGRP or its receptor for migraine prevention. These antibodies are: erenumab, fremanezumab, and galcanezumab. The antibody erenumab (commercial name – Aimovig, developed by Amgen and Novartis) selectively targets the CGRP receptor, and functions as a competitive, reversible inhibitor (Goadsby et al., 2017). It is given as a once monthly treatment to patients to prevent migraine. The antibody fremanezumab (TEV-48125) is a fully humanized IgG2 antibody, and binds to both α and β forms of CGRP ligand and thus blocks peptide binding to its receptor (Bigal et al., 2014; Walter and Bigal, 2015; Silberstein et al., 2017). The antibody is developed by Teva Pharmaceuticals and is marketed under the trade name of Ajovy. Finally, the humanized monoclonal antibody galcanezumab (Emgality) also targets the CGRP peptide and has been developed by Eli Lilly & Co. as a once monthly treatment that helps prevent future migraines (Forderreuther et al., 2018). While these humanized antibodies are showing promising results against migraine, it is important to mention here that while developing new CGRP or its receptor-targeted drugs, it is essential to consider the cardiovascular risks associated with CGRP signaling blockade (MaassenVanDenBrink et al., 2016).

## Conclusion

There is overwhelming evidence to support the theory that α-CGRP benefits the heart by decreasing Ang-II activity, increasing cardiac blood flow *via* its potent vasodilator activity, and protecting cardiomyocytes from ischemia and metabolic stress. Attempts to generate α-CGRP agonists with extended bioavailability and half-life are showing promising results that significantly reverse the adverse effects of hypertension and heart failure in animal models. Thus, α-CGRP, either in its native or a modified form, may have therapeutic benefits to patients suffering from cardiac diseases in near future.

## Author Contributions

AK wrote the manuscript. JP and DD reviewed, edited, and approved the final version of the manuscript.

### Conflict of Interest Statement

The authors declare that the research was conducted in the absence of any commercial or financial relationships that could be construed as a potential conflict of interest.

## Abbreviations

AC: Adenylate cyclase
Ang-II: Angiotensin II
BP: Blood pressure
CGRP: Calcitonin gene-related peptide
CLR: Calcitonin receptor-like receptor
DOC: Deoxycorticosterone
DRG: Dorsal root ganglia
I/R injury: Ischemia/reperfusion injury
KO: Knock-out
L-NAME: N-nitro-L-arginine methyl ester
LV: Left ventricle
NO: Nitric oxide
PKA: Protein kinase A
RAMP: Receptor activity modifying protein
RCP: Receptor component protein
SN-salt: Subtotal nephrectomy-salt
TAC: Transverse aortic constriction
WT: Wild-type.
