# Supplementary material for: Protective Role of α-Calcitonin Gene-Related Peptide in Cardiovascular Diseases
Source: Front Physiol. 2019 Jul 2;10:821. doi: 10.3389/fphys.2019.00821 (PMC6614340; doi:10.3389/fphys.2019.00821)
Supplement: Supplementary file 1 [file Table_1.pdf]

Table 1: Role of α-CGRP in various animal model of cardiovascular diseases

Disease	Animal model	Pathological /molecular changes and CGRP effects	References
Heart Failure	α-CGRP KO mice with Transacortic constriction (TAC) surgery	α-CGRP level increased in a compensatory manner in response to heart failure (in TAC WT-mice, CGRP release initially increased that decreased in later stage)<br>TAC hearts in α-CGRP KO mice have decreased FS and EF, and display increased fibrosis, necrosis, apoptosis, and inflammation.	Li et al., 2013
	Rats with ascending aortic banding	Increased expression of RAMP1 mRNA and protein in atria and ventricles.	Cueille et al, 2002
	Isoprenaline-induced HF in rat	Isoprenaline decreases CGRP production, and induces collagen deposition in heart and enhaces cardiac remodeling.	Li et al., 2010
	α-CGRP KO mice	Increased systolic blood pressure and arterial pressure in α-CGRP KO mice	Gangula et al., 2000
	Rat with left coronary artery ligation	CGRP induces dilation of blood vessels *in vitro* isolated from CHF rats	Bergdahl et al., 1999
Hypertension	DOC-salt–induced hypertensive rat	Elevated levels of CGRP in the spinal cord and α-CGRP mRNA in the DRG neurons<br>α-CGRP acts as a compensatory depressor to attenuate the rise in BP in DOC-salt rat	Supowit et al., 1995
	Subtotal nephrectomy -induced hypertensive rat	α-CGRP acts as a compensatory depressor to attenuate the rise in BP	Supowit et al., 1998
	L-NAME-induced hypertension during pregnancy	α-CGRP acts as a compensatory depressor to attenuate the rise in BP and infusion of the antagonist α-$CGRP_{8-37}$ increases the elevated blood pressure	Gangula et al., 1997
	α-CGRP-KO mice	α-CGRP regulate BP under normal physiological condition as BP and mean arterial pressure were significantly higher in α-CGRP-KO mice compared to wild-type mice<br>An increase in the activity of the Plasma RAAS is observed	Gangula et al., 2000<br>Li et al., 2004
	Ang-II induced hypertension	α-CGRP infusion significantly reduces the BP in hypertensive rats,<br>Daily administration of α-CGRP analogue (acylated - α-CGRP) significantly reverse the renal, vascular, and cardiac damage in hypertensive mice induced by Ang-II	Fujioka et al, 1991<br>Aubdool et al, 2017
Ischemia	Rats with ischemia reperfusion injury	α-CGRP administration significantly reversed myocardial I/R injury and decreases infarct size in heart.	Wolfrum et al, 2005
	α-CGRP KO mice with I/R injury	Deletion of α-CGRP makes the heart more vulnerable to I/R injury due to increased oxidative stress and inflammation	Huang et al, 2008
	Rats with ischemia reperfusion injury	Intravenous infusion of CGRP 10 min before occlusion until the end of reperfusion significantly reduces the infarcted size in heart	Wu et al, 2001
